# Establishing reference values for the pediatric abdominal aorta in MRI

**DOI:** 10.1007/s00330-025-11893-7

**Published:** 2025-08-06

**Authors:** Corona Metz, Anne Pohrt, Katja Glutig, Matthias Stephan Anders, Simon Veldhoen

**Affiliations:** 1 Charité—Universitätsmedizin Berlin, Corporate Member of Freie Universität Berlin and Humboldt-Universität zu Berlin, Pediatric Radiology, Berlin, Germany; 2 Charité—Universitätsmedizin Berlin, Corporate Member of Freie Universität Berlin and Humboldt-Universität zu Berlin, Institute of Biometry and Clinical Epidemiology, Berlin, Germany

**Keywords:** Reference values, Aorta, Abdominal, Magnetic resonance imaging, Pediatrics

## Abstract

**Objectives:**

Reference values for the diameter of the thoracic aorta exist for children across all diagnostic imaging procedures, but not for the abdominal aorta. To our knowledge, magnetic resonance imaging (MRI) based reference values for the pediatric abdominal aorta have not yet been published. The aim of this study was to determine reference values for the aortic diameter in a pediatric population using MRI.

**Materials and methods:**

150 children (mean age, 9.1 ± 5.6 years) without history or clinical signs of cardiovascular disease underwent MRI, including a three-dimensional stack of stars gradient-recalled-echo sequence with fat-saturation after intravenous contrast administration. Aortic diameters at four levels (diaphragm, celiac trunk, renal arteries, superior to the aortic bifurcation) were measured by two radiologists. Interrater reliability was analyzed using the intraclass correlation coefficient (ICC). Least squares regression models were used to relate aortic diameter to body surface area (BSA). Three model sets were evaluated with the Akaike information criterion to identify the best fit. After determining the optimal model, the influence of sex on diameter measurements was examined. Predicted diameter formulas were derived from the slope estimates, and mean squared error estimates were used to calculate *z*-scores.

**Results:**

Interrater reliability between the aortic diameter measurements was excellent (ICC, 0.98–0.99). The polynomial regression of the natural logarithm was identified being the best model for all aortic measurements.

**Conclusion:**

Reference diameters for the abdominal aorta in children were determined considering BSA and sex. Data from a healthy population provides a valuable reference for clinical evaluation.

**Key Points:**

***Question****MRI-based reference values for the pediatric abdominal aorta have not yet been published*.

***Findings****Interrater reliability between the aortic diameter measurements was excellent, and natural logarithmic polynomial regression optimally models the aortic diameter to body surface area*.

***Clinical relevance****Establishing reference values for the pediatric abdominal aorta is essential for diagnosing and monitoring cardiovascular disease. This study provides normative graphs and tables to assess aortic diameters in relation to body surface area in clinical routine*.

**Graphical Abstract:**

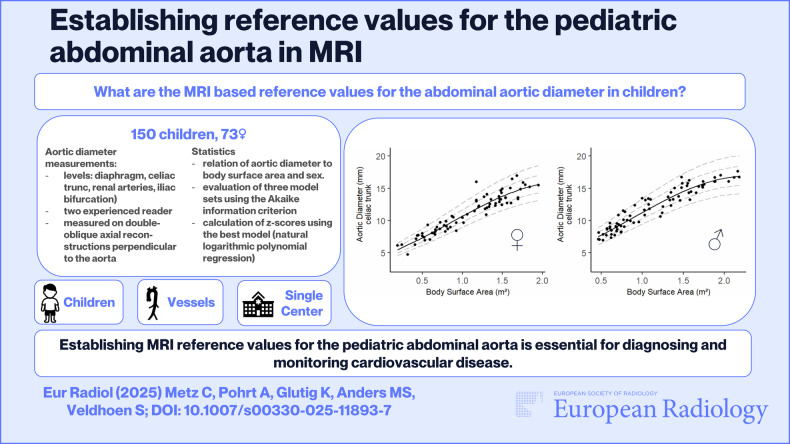

## Introduction

In children, congenital or acquired diseases can be associated with changes in the diameter of the abdominal aorta. While reference values for the diameter of the thoracic aorta are available for practically all diagnostic imaging procedures [1–4], the data available for the abdominal aorta are sparse and based on very few studies. Existing studies provide reference values for the diameter of the abdominal aorta based on ultrasound examinations or computed tomography (CT) [5–7]. While ultrasound examination of the abdominal aorta is an examiner-dependent method and regularly does not allow reliable visualization of the entire abdominal aorta, particularly in small children due to bowel gas and limited compliance, CT is associated with undesirable radiation exposure.

In pediatric radiology, MRI is frequently used for diagnostic imaging of the abdomen for various medical issues due to its radiation-free nature, also for the clarification of unclear symptom constellations to rule out serious diseases. To our knowledge, reference values for the pediatric abdominal aorta based on MRI measurements have not yet been published. Electrocardiogram (ECG) triggered magnetic resonance angiography (MRA) of the abdominal aorta is not regularly performed in children, especially if the aorta is not in diagnostic focus: the examination time and anesthesia, which is regularly required at a young age, is considerably extended by the additional preparation (application of electrodes etc.) and execution of this technique.

Nevertheless, it is of utmost importance to correctly assess the diameter of the pediatric abdominal aorta in standard MRI to reliably evaluate hypoplasia or dilatation, which can be part of syndromic diseases [8, 9]. The aim of this study was to determine normal values for the aortic diameter in children using MRI in a population of children and adolescents with no history or clinical signs of cardiovascular disease.

## Materials and methods

### Institutional review

The study was approved by the institutional review board (*Charité—Universitätsmedizin Berlin*).

### Study sample and design

In this retrospective study, the normal diameter of the aorta at different levels of children in MRI was investigated. The inclusion criterion for children evaluated between 2021 and 2024 was the successful acquisition of a T1-weighted contrast-enhanced isotropic three-dimensional (3D) stack-of-stars gradient-echo sequence (StarVIBE) with fat-saturation for a clinical indication covering the entire abdomen from the diaphragm to the iliac bifurcation. The sequence was acquired during free-breathing using the following sequence parameters: Field of view (FOV) options of 244 × 244 mm², 300 × 300 mm² and 340 × 340 mm² were selected based on patient size; voxel size = 1.4 × 1.4 × 3.0 mm³; repetition time (TR) = 3.5 ms; echo time (TE) = 1.42 ms; spokes = 660; flip angle = 9°; bandwidth = 830 Hz/pixel; acquisition time, 3.59 min.

Exclusion criteria were a history or clinical evidence of cardiovascular disease, absence of a StarVIBE sequence after contrast agent application covering the entire abdomen, significant artefacts affecting the aorta, and patient age > 18 years. If patients underwent multiple MRI scans, only the first examination was included in the study.

Based on these criteria, 150 of 651 screened MRI examinations of the abdomen were included in the study. The body surface area (BSA) for each patient was calculated by using the Mosteller formula [10, 11]:

$${{B}}{{S}}{{A}}\,[{{{m}}}^{2}]=\surd ({{w}}{{e}}{{i}}{{g}}{{h}}{{t}}\,({{k}}{{g}})\times {{h}}{{e}}{{i}}{{g}}{{h}}{{t}}\,({{c}}{{m}})/3600)$$

**Fig. 1:**
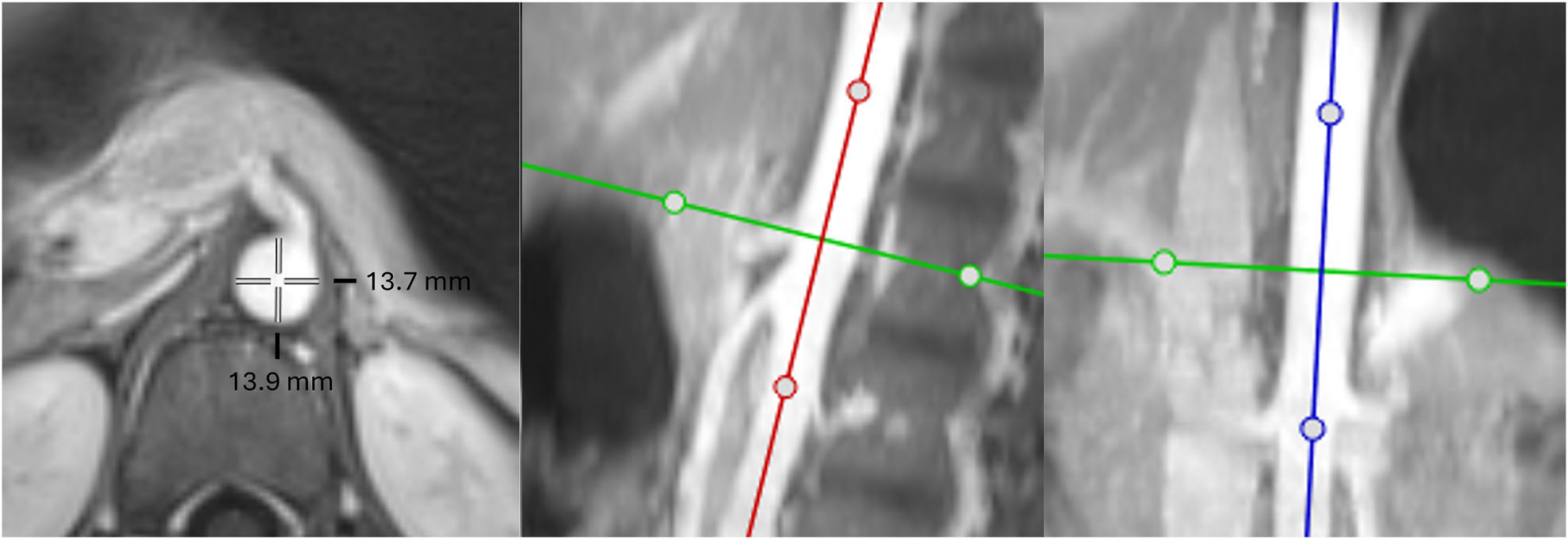
Aortic diameter measurement. Determination of the aortic diameter at the level of the celiac trunk using double-oblique reconstructions created perpendicular to the aorta

All study examinations were performed on a 3Tesla scanner (Skyra fit, Siemens Healthcare) using an 18-channel flex body coil. Gadovist^®^ (Bayer Vital GmbH) was used as an intravenous contrast agent at 0.1 mL per kilogram body weight. Images were acquired in axial orientation, and multiplanar reformations were created using a commercially available picture archiving and communication system (Phoenix-PACS) on a clinical workstation. Multiplanar reformations were double-oblique reconstructions obtained perpendicular to the aorta (Fig. 1).

The mean diameter at each level was determined by averaging the anteroposterior and lateral diameter measurements. The aortic levels at which measurements were obtained were as follows: abdominal aorta at the diaphragm (at the level of the median arcuate ligament), abdominal aorta at the celiac trunk, abdominal aorta at the level of the renal arteries, and abdominal aorta superior to the aortic bifurcation. Pseudonymized and randomized images from all study subjects were independently read by two radiologists (S.V., a subspecialist in pediatric radiology with twelve years of experience, and C.M., a specialist in radiology with six years of experience, two years of experience in pediatric radiology). Each radiologist independently created the double-oblique multiplanar reconstructions from the original axial datasets and used electronic calipers to measure anteroposterior and lateral inner-to-inner diameters that were used to determine the mean diameter (Fig. 1).

Subsequent analyses were performed using the mean diameter from the anteroposterior and lateral measurements and the area of the aorta at the respective level.

### Statistics

Although the study was exploratory in nature and not designed for formal hypothesis testing, a preliminary sample size estimation was performed to ensure adequate coverage of the pediatric age and body size spectrum. Using a two-sided 95% confidence interval and assuming normal distribution, the expected standard deviation (SD) for aortic diameter was derived from a retrospective review of existing abdominal MRI datasets. Based on these values, two calculations were conducted in nQuery (Version 9.1.0.0, Statistical Solutions Ltd) to estimate the range of required sample sizes, depending on the distance from the mean to the confidence interval boundaries.

To allow for clinically meaningful subgroup analyses, the study population was divided into age groups based on developmental stages in childhood and in alignment with groupings used in existing literature.

The interrater reliability of the diameter measurements of the two readers was evaluated by calculating the intraclass correlation coefficient (ICC) [12]. To describe the relationship between the aortic diameter at a particular level, which is the dependent variable, and the independent variables BSA and sex, ordinary least squares regression models were fitted. Three sets of models that were cited in Hegde et al [5] and were initially described in studies modeling the aortic diameter measured with echocardiography [13, 14] were evaluated by using Akaike information criterion (AIC) to determine the functional form with the best fit:

Model 1

$${{D}}{{i}}{{a}}{{m}}{{e}}{{t}}{{e}}{{r}}={\beta }_{0}+{\beta }_{1}\cdot BSA+{\beta }_{2}\cdot BS{A}^{2}+{\beta }_{3}\cdot BS{A}^{3}$$

Model 2

$${{{{\rm{l}}}}{{{\rm{o}}}}{{{\rm{g}}}}}_{e}({{D}}{{i}}{{a}}{{m}}{{e}}{{t}}{{e}}{{r}})={\beta }_{0}+{\beta }_{1}\cdot BSA+{\beta }_{2}\cdot BS{A}^{2}+{\beta }_{3}\cdot BS{A}^{3}$$

Model 3

$${{{{\rm{l}}}}{{{\rm{o}}}}{{{\rm{g}}}}}_{e}({{D}}{{i}}{{a}}{{m}}{{e}}{{t}}{{e}}{{r}})={\beta }_{0}+{\beta }_{1}\cdot {{{l}}{{o}}{{g}}}_{e}(BSA)$$

${\beta }_{0}$ represents the intercept, while ${\beta }_{1}$- ${\beta }_{3}$ are the slopes estimated by the model for linear, quadratic, and cubic terms. BSA is used to predict the aortic diameter or its logarithm. Once the optimal model had been identified, the influence of sex was investigated by incorporating a sex main effect into the model.

From the slope estimates derived from these models, formulas were specified for the predicted diameters, and estimates of the mean squared error (MSE) were obtained, allowing the *z*-scores to be calculated. The analyses were performed using R Statistical Software (v4.1.2; R Core Team 2021). *p*-values < 0.05 were considered statistically significant.

## Results

### Patient characteristics

Of the 150 patients included in the study, 73 were female. The age range was 0 to 18 years (mean 9.1 ± 5.6 years; median 9 years). MRI examinations were clinically indicated for the following reasons: hemato-oncological assessments (*n* = 74), such as tumor staging or imaging in sickle cell disease; urogenital indications (*n* = 12), including duplex kidney or pyelonephritis; gastrointestinal imaging (*n* = 29), such as for inflammatory bowel disease; evaluation of solid abdominal organs (*n* = 32), including assessment of liver lesions or pancreatic cysts; and post-transplantation follow-up (*n* = 3).

BSA ranged from 0.2 m^2^ to 2.2 m^2^ (mean, 1.1 ± 0.5 m^2^; median, 1.1 m^2^). There was no significant difference between the sexes regarding age, BSA or aortic diameter measurements. Patient data are summarized in Table 1.

**Table 1:** Study population

	Female	Male	*p*
*n*	73	77	
Mean age ± SD in years	9.6 ± 5.7	9.4 ± 5.5	0.87
Mean height ± SD in cm	131.2 ± 33.1	135.1 ± 34.1	0.48
Mean weight ± SD in kg	34.0 ± 19.6	35.7 ± 22.3	0.63
Number per age group (%)			1.00
[0–2] years	8 (11.0)	9 (11.7)	
[2–4] years	9 (12.3)	8 (10.4)	
[4–6] years	10 (13.7)	12 (15.6)	
[6–11] years	13 (17.8)	14 (18.2)	
[11–15] years	17 (23.3)	17 (22.1)	
[15–19] years	16 (21.9)	17 (22.1)	
Mean diameter diaphragm ± SD in mm	11.7 ± 3.1	12.4 ± 3.1	0.16
Mean diameter celiac trunk ± SD in mm	11.2 ± 3.0	12.0 ± 3.1	0.14
Mean diameter renal arteries ± SD in mm	9.3 ± 2.7	9.9 ± 2.9	0.18
Mean diameter aortic bifurcation ± SD in mm	9.0 ± 2.8	9.7 ± 3.1	0.13
Mean area diaphragm ± SD in mm^2^	114.27 ± 55.6	128.0 ± 61.9	0.16
Mean area celiac trunk ± SD in mm^2^	105.3 ± 52.7	119.5 ± 59.5	0.13
Mean area renal arteries ± SD in mm^2^	73.6 ± 39.5	83.1 ± 45.7	0.17
Mean area aortic bifurcation ± SD in mm^2^	69.6 ± 39.4	81.3 ± 48.3	0.11

Description of the patient characteristics relevant to the study

### Aortic diameter measurements

The interrater reliability between the aortic diameter measurements made by the two readers was excellent, as indicated by the ICCs, which ranged from 0.98 to 0.99 (Table 2).

**Table 2:** Interrater reliability of the aortic diameter measurement

Aortic level	Intraclass correlation coefficient
Mean diameter diaphragm	0.99
Mean diameter celiac trunk	0.99
Mean diameter renal arteries	0.99
Mean diameter aortic bifurcation	0.98
Area diaphragm	0.99
Area celiac trunk	0.99
Area renal arteries	0.99
Area aortic bifurcation	0.98

Intraclass correlation of mean diameter and area measurements at different aortic levels as measured by two radiologists

In our study the anteroposterior diameter differed significantly from the lateral diameters at all levels (mean anteroposterior diameter: diaphragm, 11.8 mm; celiac trunk, 11.3 mm; renal arteries, 9.4 mm; aortic bifurcation, 9.1 mm; mean lateral diameter: diaphragm, 12.3 mm; celiac trunk, 11.6 mm; renal arteries, 9.9 mm; aortic bifurcation, 9.7 mm; all *p* < 0.005).

### Regression models

The best model of the models used in Hegde et al [5] was identified by comparing the AIC and turned out to be the polynomial regression model of the natural logarithm of the diameter (Model 2) for all levels. AIC was the smallest for this model at all levels. The results when using the mean aortic diameter tended to be slightly better than when using the aortic area.

**Fig. 2:**
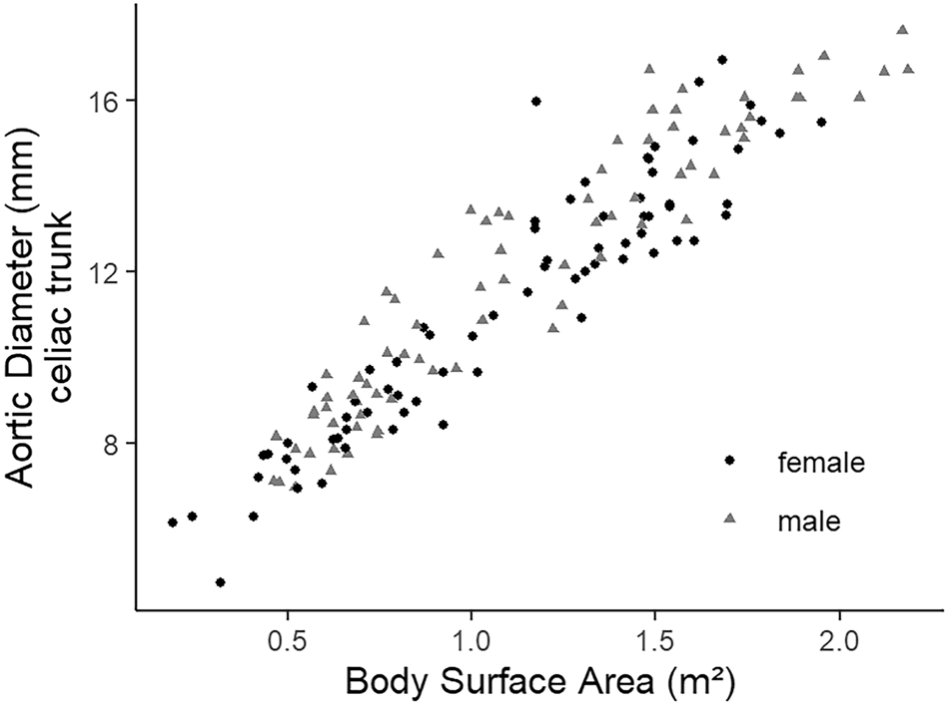
Relation of the aortic diameter to the body surface area. A positive correlation between the mean aortic diameter at the level of the celiac trunk and the body surface area in male and female children

Adding sex to the modeling equation was significant for all levels (*p* < 0.05). Figure 2 shows the correlation of the measured aortic diameter to BSA for both sexes. Age is highly correlated with BSA, with a Spearman’s correlation coefficient of 0.92.

The formulas with the AIC, square root of the mean squared error (sqrtMSE), and the proportion of variance (*R*^2^), for the final models, which can be used to calculate the predicted diameter of the aorta at various levels, are shown in Table 3. The *z*-score at a particular aortic level could then be calculated in the following manner:

$${{p}}{{r}}{{e}}{{d}}{{i}}{{c}}{{t}}{{e}}{{d}}\,{{v}}{{a}}{{l}}{{u}}{{e}}\,{{f}}{{r}}{{o}}{{m}}\,{{B}}{{S}}{{A}}-{{a}}{{c}}{{t}}{{u}}{{a}}{{l}}\,{{v}}{{a}}{{l}}{{u}}{{e}}|/{{s}}{{q}}{{r}}{{t}}{{M}}{{S}}{{E}}\,{{a}}{{t}}\,{{t}}{{h}}{{a}}{{t}}\,{{B}}{{S}}{{A}}$$

**Table 3:** Calculation of the aortic diameter

	Formula	AIC	sqrtMSE	*R*^2^
Diameter diaphragm	ln(MD) = 1.573 + 0.047 · sex(male) + 1.072 · BSA -0.229 · BSA² -0.002 · BSA³	−314.390	0.082	0.909
Diameter celiac trunk	ln(MD) = 1.506 + 0.052 · sex(male) + 1.098 · BSA -0.23 · BSA² -0.003 · BSA³	−295.421	0.087	0.903
Diameter renal arteries	ln(MD) = 1.214 + 0.053 · sex(male) + 1.17 · BSA -0.176 · BSA² -0.033 · BSA³	−262.595	0.097	0.904
Diameter aortic bifurcation	ln(MD) = 1.093 + 0.061 · sex(male) + 1.317 · BSA -0.26 · BSA² -0.013 · BSA³	−247.900	0.102	0.907
Area diaphragm	ln(MD) = 2.9 + 0.093 · sex(male) + 2.153 · BSA -0.465 · BSA² -0.002 · BSA³	−105.753	0.163	0.908
Area celiac trunk	ln(MD) = 2.771 + 0.104 · sex(male) + 2.189 · BSA -0.457 · BSA² -0.007 · BSA³	−86.531	0.174	0.903
Area renal arteries	ln(MD) = 2.183 + 0.096 · sex(male) + 2.31 · BSA -0.31 · BSA² -0.079 · BSA³	−39.390	0.204	0.896
Area aortic bifurcation	ln(MD) = 1.948 + 0.114 · sex(male) + 2.596 · BSA -0.476 · BSA² -0.038 · BSA³	−31.549	0.209	0.903

Formulas to calculate the predicted diameter on a natural logarithm scale

*AIC* Akaike information criterion, *sqrtMSE* square root of the mean squared error, *R*² proportion of variance explained by the model), *ln* natural logarithm, *MD* mean diameter

Exemplarily at the celiac trunk level *(using* Table 3*)*:

$$	 |exp (1.51+0.05\cdot {{s}}{{e}}{{x}}({{m}}{{{\rm{a}}}}{{{\rm{l}}}}{{{\rm{e}}}})+1.10\cdot BSA-0.23\cdot BS{A}^{2} \\ 	 -0.003\cdot BS{A}^{3})-({{a}}{{c}}{{t}}{{u}}{{a}}{{l}}\,{{c}}{{e}}{{l}}{{i}}{{a}}{{c}}\,{{d}}{{i}}{{a}}{{m}}{{e}}{{t}}{{e}}{{r}})|/{{{s}}{{q}}{{r}}{{t}}{{M}}{{S}}{{E}}}_{{{c}}{{e}}{{l}}{{i}}{{a}}{{c}}}$$

**Fig. 3:**
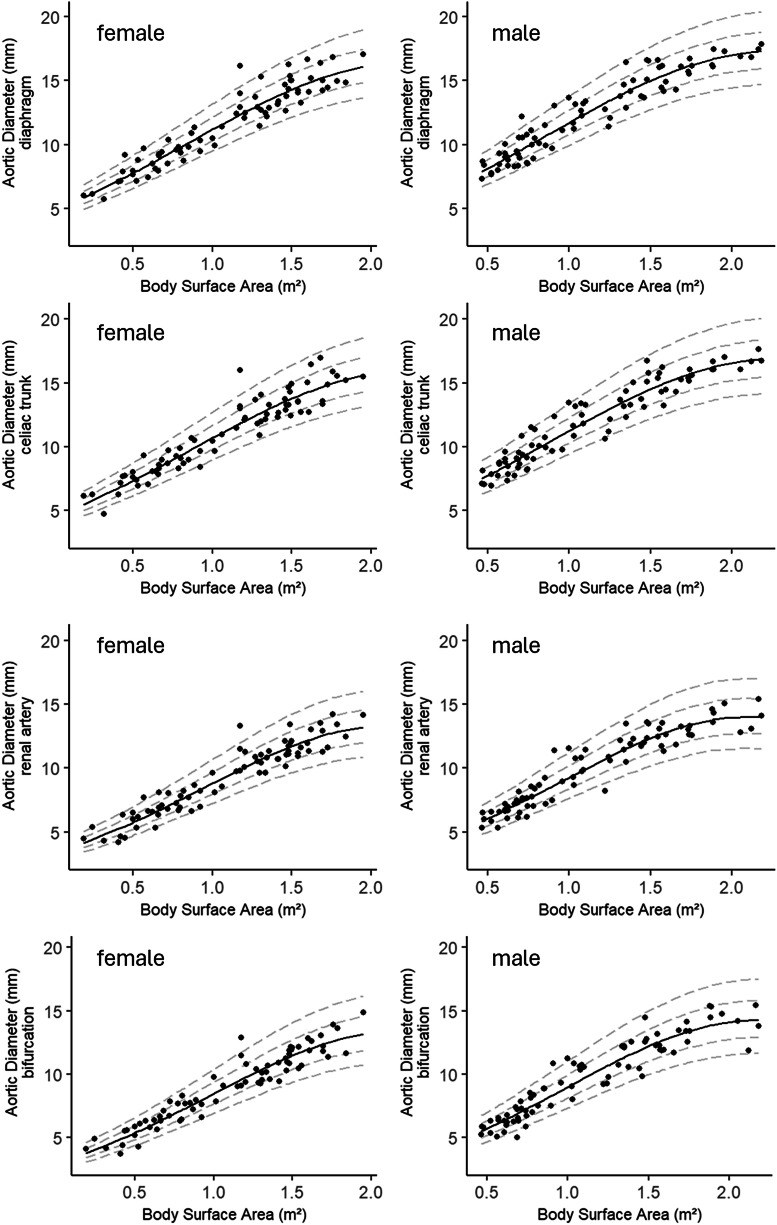
Standard values of the pediatric abdominal aorta in MRI. Mean aortic diameter at all levels versus body surface area in male and female children. *z*-scores (dashed lines) indicating the number of standard deviations (1 standard deviation (SD), inner dashed line; 2 SD, outer dashed lines) above and below the mean value (continuous line)

The *z*-scores follow an approximately normal distribution, with a mean of 0 and a SD of 1. *z*-scores indicate how many SDs an observation deviates from the predicted mean. This information is also presented in graphical form, as shown for each level in Fig. 3, so that no additional calculations are required for individual measured values. In addition to the graphical representation of aortic diameters, Table 4 provides the corresponding reference values stratified by BSA, enabling straightforward interpretation of individual measurements.

**Table 4:** Standard values of the pediatric abdominal aorta in MRI

BSA (in m^2^)	Age (in years)	Diaphragm (in mm)	Celiac trunc (in mm)	Renal arteries (in mm)	Aortic bifurcation (in mm)
Female					
0.30	~ 0–1	6.8 (5.8–8)	6.8 (5.8–8.1)	5.0 (4.1–6)	4.6 (3.8–5.6)
0.50	~ 1–3	8.2 (7–9.6)	8.2 (6.9–9.7)	6.1 (5–7.3)	5.7 (4.7–7)
0.75	~ 3–6	9.9 (8.5–11.6)	9.9 (8.4–11.8)	7.6 (6.3–9.2)	7.3 (6–8.9)
1.00	~ 6–10	11.7 (10–13.8)	11.7 (9.9–13.9)	9.3 (7.7–11.2)	9 (7.4–11)
1.25	~ 10–13	13.4 (11.5–15.8)	13.4 (11.3–15.9)	10.9 (9–13.2)	10.7 (8.8–13.1)
1.50	~ 13–15	15 (12.8–17.6)	15 (12.6–17.8)	12.3 (10.2–14.9)	12.2 (10–14.9)
1.75	~ 15–17	16.2 (13.8–19)	16.2 (13.7–19.2)	13.4 (11.1–16.2)	13.4 (11–16.3)
2.00	~ 17–19	17 (14.5–20)	17 (14.3–20.2)	14 (11.6–16.9)	14.1 (11.6–17.2)
Male					
0.30	~ 0–1	6.5 (5.6–7.6)	6.1 (5.5–7.7)	4.7 (3.9–5.7)	4.3 (3.5–5.3)
0.50	~ 1–3	7.8 (6.6–9.1)	7.4 (6.6–9.2)	5.8 (4.8–7)	5.4 (4.4–6.6)
0.75	~ 3–6	9.5 (8.1–11.1)	9 (8–11.2)	7.2 (6–8.7)	6.9 (5.6–8.4)
1.00	~ 6–10	11.2 (9.5–13.1)	10.7 (9.4–13.3)	8.8 (7.3–10.6)	8.5 (6.9–10.4)
1.25	~ 10–13	12.8 (10.9–15.1)	12.3 (10.8–15.2)	10.3 (8.6–12.5)	10.1 (8.2–12.3)
1.50	~ 13–15	14.3 (12.2–16.8)	13.8 (12.1–16.9)	11.7 (9.7–14.2)	11.5 (9.4–14)
1.75	~ 15–17	15.5 (13.2–18.1)	14.9 (13–18.3)	12.7 (10.5–15.4)	12.6 (10.3–15.4)
2.00	~ 17–19	16.2 (13.8–19)	15.7 (13.7–19.2)	13.2 (11–16)	13.3 (10.9–16.2)

Values represent the average of the anteroposterior and lateral aortic diameter derived from MRI measurements at predefined anatomical levels. Values in parentheses represent the model-based 2.5th–97.5th percentile. The typical age is provided for orientation only—BSA is the decisive reference parameter

*BSA* body surface area

## Discussion

To ascertain whether a pathological aortic diameter is present, the normal values of the aortic diameter must be known. In this study, the diameter of the abdominal aorta was therefore determined at different levels in children of different sizes and both sexes. Age was not included in the models for the aortic diameters, because it is highly correlated with the main predictor, BSA.

Aortic diameter measurements should be taken at standardized anatomical landmarks perpendicular to the axis of blood flow [2, 14]. Three-dimensional CT and isotropic MR imaging datasets are ideal for generating multiplanar reconstructions perpendicular to the aorta, ensuring accurate diameter measurements. Due to the physiological oblique course of the aorta, axial reconstructions can lead to errors in measuring the true diameter, which is why double-oblique reconstructions are recommended [15]. Since the aorta is not perfectly round [2], we recommend assessing its mean diameter by calculating the average of the anteroposterior and lateral diameter measurements at a given level. When calculating the area, both diameters—the anteroposterior and lateral—are used anyway. However, based on the results of this study, we suggest using the mean aortic diameter instead of the aortic area at a specific level. This approach was chosen because it is simple, reproducible, and less affected by asymmetry in vessel shape than single-plane measurements. While alternatives such as perimeter-based diameters exist, they typically require complex segmentation steps and are less established in clinical imaging.

Unlike Hegde et al [5], we decided in favor of the AIC instead of *R*^2^, as AIC is an estimator for the prediction error, which addresses both the risk of overfitting and the risk of underfitting. However, an additional analysis using *R*^2^ also showed that Model 2 performed best.

In addition to the three listed models, which originate from other publications, an attempt was made to find a better model for the aortic diameter and BSA using fractional polynomials. Fractional polynomials are a class of functions for diameter where BSA can enter with two different exponents. With a dedicated package in the software R, a set of exponents that best model the diameter is selected in a backward elimination process. These models were not significantly better than Model 2 (results not shown).

Published ultrasound- and CT-based reference values for the diameter of the abdominal aorta in children show a similar trend and a similar correlation with body size metrics such as BSA and age [5, 7]. Nevertheless, slight systematic differences in absolute values can be expected due to the limitations of direct cross-modality comparison by differing acquisition techniques and a lack of intraindividual comparison. This reinforces the value of establishing modality-specific reference ranges to support accurate clinical interpretation.

Finally, recognizing that the widely used DuBois formula tends to underestimate BSA in children with a BSA below 0.7 m², we opted for the Mosteller formula in this study to improve accuracy in the pediatric population [10, 14, 16, 17].

### Limitations

A limitation of our study is the relatively small sample size in certain age subgroups, which may affect the generalizability of reference values for those specific ranges. Additionally, as detailed information on the ethnic background of participants was not systematically collected, potential variations in aortic dimensions related to ethnic differences could not be evaluated. Furthermore, undiagnosed cardiovascular disease cannot be definitively excluded, as no formal cardiovascular screening was performed; however, all included children had no history or clinical signs of cardiovascular disease.

As with most MRI examinations of the abdomen in children, the sequence used in this study was acquired without ECG triggering and during free-breathing. These acquisition settings can lead to mild blurring artefacts, particularly in the region of the abdominal aorta near the diaphragm, and may potentially result in a slight overestimation of the vessel diameter. However, to ensure reliable measurements, all examinations with pronounced artefacts—such as significant motion, ghosting, or blurring—were excluded from the analysis. Moreover, the excellent interrater reliability observed in our study supports the robustness and reproducibility of the measurements despite the aforementioned limitations. Importantly, we aimed to establish reference values based on standard clinical sequences routinely used in pediatric abdominal MRI, thus ensuring the practical applicability of our results in everyday clinical practice.

## Conclusion

The normal diameter of the abdominal aorta in children was determined for different aortic levels, with consideration given to BSA and sex. Establishing reference values for the abdominal aorta in children is essential for the diagnosis and monitoring of cardiovascular and other diseases.

## Abbreviations

AIC: Akaike information criterion
BSA: Body surface area
CT: Computed tomography
ECG: Electrocardiogram
ICC: Intraclass correlation coefficient
MRI: Magnetic resonance imaging
SD: Standard deviation
sqrtMSE: Mean squared error
